# Functional Neural Changes after Low-Frequency Bilateral Globus Pallidus Internus Deep Brain Stimulation for Post-Hypoxic Cortical Myoclonus: Voxel-Based Subtraction Analysis of Serial Positron Emission

**DOI:** 10.3390/brainsci10100730

**Published:** 2020-10-13

**Authors:** Myung Ji Kim, So Hee Park, Kyoung Heo, Jin Woo Chang, Joong Il Kim, Won Seok Chang

**Affiliations:** 1 Department of Neurosurgery, Brain Research Institute, College of Medicine, Yonsei University, Seoul 03722, Korea; KMJ8686@yuhs.ac (M.J.K.); SHPARK123@yuhs.ac (S.H.P.); JCHANG@yuhs.ac (J.W.C.); 2 Department of Neurology, College of Medicine, Yonsei University, Seoul 03722, Korea; KHEO@yuhs.ac; 3 Future Medicine Division, Korea Institute of Oriental Medicine, Daejeon 34054, Korea

**Keywords:** deep brain stimulation (DBS), globus pallidum internus (Gpi), Lance–Adams syndrome (LAS), PET/MRI co-registration, post hypoxic myoclonus (PHM), voxel-based subtraction analysis

## Abstract

Post-hypoxic myoclonus (PHM) and Lance–Adams syndrome (LAS) are rare conditions following cardiopulmonary resuscitation. The aim of this study was to identify functional activity in the cerebral cortex after a hypoxic event and to investigate alterations that could be modulated by deep brain stimulation (DBS). A voxel-based subtraction analysis of serial positron emission tomography (PET) scans was performed in a 34-year-old woman with chronic medically refractory PHM that improved with bilateral globus pallidus internus (Gpi) DBS implanted three years after the hypoxic event. The patient required low-frequency stimulation to show myoclonus improvement. Using voxel-based statistical parametric mapping, we identified a decrease in glucose metabolism in the prefrontal lobe including the dorsolateral, orbito-, and inferior prefrontal cortex, which was suspected to be the origin of the myoclonus from postoperative PET/magnetic resonance imaging (MRI) after DBS. Based on the present study results, voxel-based subtraction of PET appears to be a useful approach for monitoring patients with PHM treated with DBS. Further investigation and continuous follow-up on the use of PET analysis and DBS treatment for patients with PHM are necessary to help understanding the pathophysiology of PHM, or LAS.

## 1. Introduction

Post-hypoxic myoclonus (PHM) is a rare disorder occurring in survivors of profound hypoxic episodes following cardiopulmonary resuscitation [1]. PHM commonly manifests as multifocal, generalized muscle jerks that increase during movement and usually disappear with body and limb relaxation [2]. Chronic PHM, or Lance–Adams Syndrome (LAS), develops days or weeks after hypoxia when the patient has regained consciousness [3]. Myoclonus may be positive, defined as sudden involuntary muscular contracture, or negative, characterized by interruptions of tonic activity of voluntary muscles [4,5,6]. Myoclonus is classified as cortical, subcortical, or spinal, according to the presumable generator site [7]. Patients with cortical myoclonus usually show involuntary movements consisting of both positive and negative myoclonus [8]. The pathophysiology of PHM remains unknown. Recent imaging studies have suggested that modalities such as cerebral single-photon emission computed tomography (SPECT) or positron emission tomography (PET) may provide insights into disease pathophysiology. There is some heterogeneity regarding PET and SPECT findings, including hypermetabolism in the ventrolateral thalamus and hypometabolism in the frontal lobes, indicating a heterogeneous mechanism of myoclonus in patients with LAS [9,10]. Treatment for chronic myoclonus requires a combination of multiple antiepileptic medications, such as levetiracetam, piracetam, clonazepam, and valproate [11,12]. Medical treatment with antiepileptic drugs may not satisfactorily improve the myoclonus, and deep brain stimulation (DBS) has been suggested in patients with medically-refractory chronic PHM [4,13,14,15,16]. However, DBS either targets the thalamus or uses high-frequency stimulation of the globus pallidus internus (Gpi). In the present study, the patient’s myoclonus responded better to low-frequency than high-frequency LAS. Therefore, functional changes after DBS were evaluated in this patient using PET/magnetic resonance imaging (MRI) co-registration and voxel-based serial PET subtraction.

## 2. Materials and Methods

### 2.1. Patient Data

**Figure 1:**
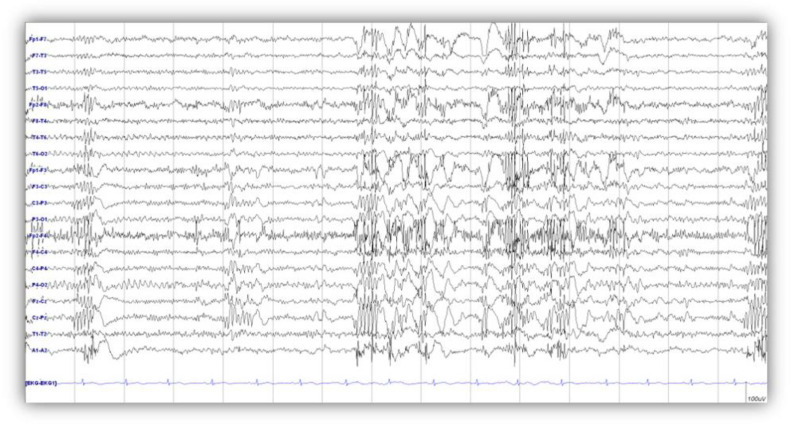
Electroencephalogram (EEG) showed very frequent bilateral, generalized, high-voltage polyspike discharges, mainly from prefrontal and frontal area, accompanied by jerking movements in the legs and slow-wave complexes with 2–4-Hertz (Hz) intervals.

A 34-year-old woman was found unconscious in deep water while snorkeling and was transported to a hospital. After successful cardiopulmonary resuscitation, she remained comatose for several days. Remarkably, she regained consciousness 14 days later. The patient’s condition improved after 1 month and she was able to follow simple commands. However, her action myoclonus aggravated, occurring at rest and worsening with movement of her hands and legs. Two months after CPR, neurological examination of the patient showed an alert, cooperative state and orientation to person, place, and time. A Mini Mental Status Examination (MMSE) yielded 29 points on a 30-point scale. She reported no family history of movement disorders. She underwent a tracheostomy that was reversed after 17 months. MRI taken two months after the hypoxic event was normal. Her electroencephalogram (EEG) showed very frequent bilateral, generalized, high-voltage polyspike discharges, mainly from prefrontal and frontal areas, accompanied by jerking movements in the legs and slow-wave complexes with 2–4-Hertz (Hz) intervals (Figure 1). She showed periodic involuntary movements of both arms and legs precipitated by action. Action myoclonus appeared in her outstretched arms and increased to myoclonic jerks during finger-to-nose movements. She could not hold a cup because of her action myoclonus (Supplementary Materials Video S1). She needed assistance to stand, which triggered negative myoclonus in her legs, and the shock-like involuntary negative myoclonus caused postural lapses (Supplementary Materials Video S2). She was unable to walk due to the myoclonus and needed a wheelchair to ambulate. The patient’s clinical features including positive and negative myoclonus with EEG findings indicated generalized cortical myoclonus. Based on her clinical examination results, LAS was diagnosed. The patient received intensive rehabilitation and antiepileptic treatment for three years after the hypoxic event. A regimen of levetiracetam (1500 mg twice a day), valproic acid (600 mg three times a day), topiramate (100 mg twice a day), and clonazepam (1 mg three times a day) provided only limited control of her rest and action myoclonus and further medication increases could not reduce the severity or frequency of her myoclonus. 

The patient provided written informed consent to participate in the study and for the publication of this report. The ethics committees (Severance Hospital Human Research Protection Center; Code: 4-2013-0795) of our institution approved the study protocol. 

### 2.2. Surgical Procedures

**Figure 2:**
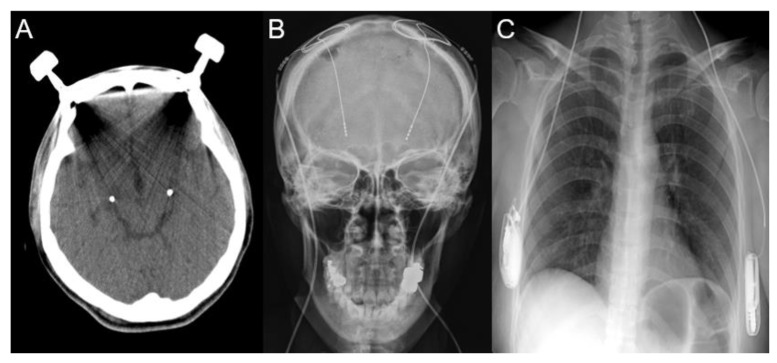
(**A**) Postoperative computed tomography (CT) scans revealed bilateral deep brain stimulation (DBS) electrodes implantation in the globus pallidum internus (Gpi) without any surgery-related complication. (**B**,**C**) 10 days after DBS, implantable pulse generators (IPGs) were implanted subcutaneously in the sub-axillary region.

**Figure 3:**
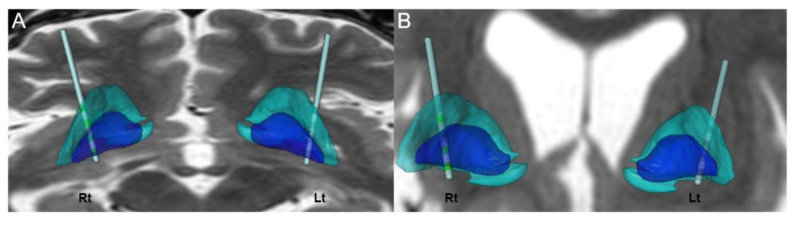
The lead analysis was performed to identify the actual locations of each contact. Blue represents the globus pallidum internus (Gpi) and blue green represents the globus pallidum externus (Gpe). Contact 0 and 1 are located within the Gpi for both electrodes. (**A**) Axial plane (**B**) Coronal plane.

Because the patient had myoclonus refractory to medication, a decision was made to perform DBS to improve her myoclonus and quality of life, three years after her hypoxic event. The decision to choose the bilateral Gpi as the target for implantation was based on our experience as well as on published data regarding the treatment of myoclonus in patients with myoclonus-dystonia (MD) with Gpi DBS [17,18,19]. The operation involved frame-based stereotactic implantations of the DBS electrodes targeting the Gpi. Preoperative stereotactic 1.5-T MRI was performed and the images transferred to a Leksell SurgiPlan (Elekta, Stockholm, Sweden). The stereotactic coordinate for Gpi localization was 20.5 mm lateral (X), 2 mm anterior (Y), 3 mm inferior (Z) to the midcommissural point (MCP). Gpi localization was verified with MRI and the Schaltenbrand atlas. The procedure was performed under local anesthesia to evaluate the effects of stimulation and possible side effects using microstimulation during microelectrode recordings (MERs) and macrostimulation through the permanent electrode. For permanent stimulation, DBS electrodes (model 3387, Medtronic, Minneapolis, MN, USA) were used. After implanting the electrodes, postoperative computed tomography (CT) scans were acquired before removing the frame (Figure 2A) and fused with preoperative MR images to identify the positions of the electrodes. After 10 days of test stimulation, implantable pulse generators (IPGs) (Activa SC, Medtronic) were implanted subcutaneously in the sub-axillary region under general anesthesia (Figure 2B,C). The lead analysis was performed with Suretune 3 (Medtronics) to identify the actual location of each contact. Figure 3 reveals that contact 0 and 1 are located within the Gpi for both electrodes.

### 2.3. Brain Image Acquisition and Preprocessing

Because the conventional MRI presented no definite structural abnormality, fluorodeoxyglucose positron emission tomography (^18^F-FDG PET) was performed three times—preoperatively, one year after DBS, and three years after DBS—to identify functional activity of the cerebral cortex after the hypoxic event and to investigate alterations that could be modulated by DBS. Postoperative PET scans were performed with DBS stimulation on. ^18^F-FDG PET images were obtained on a PET/CT scanner (Discovery STE PET/CT scanner, GE Healthcare, USA). Approximately 370 MBq of ^18^F-FDG was administered intravenously 60 min before scanning. ^18^F-FDG PET images were acquired for 10 min, and repeatedly reconstructed with CT-based attenuation correction according to the following parameters: field of view = 128 × 128, pixel size = 2.34 × 2.34 mm^2^, slices = 65, slice thickness = 2.38 mm. Brain anatomical images of high-resolution T1-weighted MRI on a 3-Tesla (T) MRI scanner (Achieva, PHILIPS Healthcare, Netherlands) were obtained using the following parameters: field of view = 256 × 256, voxel size = 0.86 × 0.86 × 1.0 mm^3^, slices = 170, echo time (TE) = 9.89 ms, repetition time (TR) = 4.6 ms, flip angle = 8°. In the image analysis, only preoperative 3-T MRI images (before DBS implantation) were used due to the potential morphological changes to the skull that may have occurred in the brain after surgery. 

All analyses of changes in preoperative and postoperative brain glucose metabolism were performed using statistical parametric mapping (SPM12, Wellcome Trust Centre for Neuroimaging, University College London, London, UK) implemented in MATLAB (version 2018b, MathWorks Inc., Natick, MA, USA). To analyze the images at spatially common coordinates of individual brains, each ^18^F-FDG PET image obtained at different time points during the follow-up period was co-registered to the MRI image using rigid-body transformation. These MRI co-registered PET images can visualize a distributed map of radioisotope uptake levels on individual brain MRIs and localize the neural activation or deactivation regions [20]. Co-registered ^18^F-FDG PET images were subsequently smoothed by convolution with an isotropic 3D Gaussian kernel of 6-mm full width at half maximum (FWHM). Finally, the image intensities of ^18^F-FDG PET were scaled in proportion to the overall mean (conventional global mean scaling) for voxel-based subtraction analysis [21].

### 2.4. Voxel-Based Subtraction Analysis of PET

**Figure 4:**
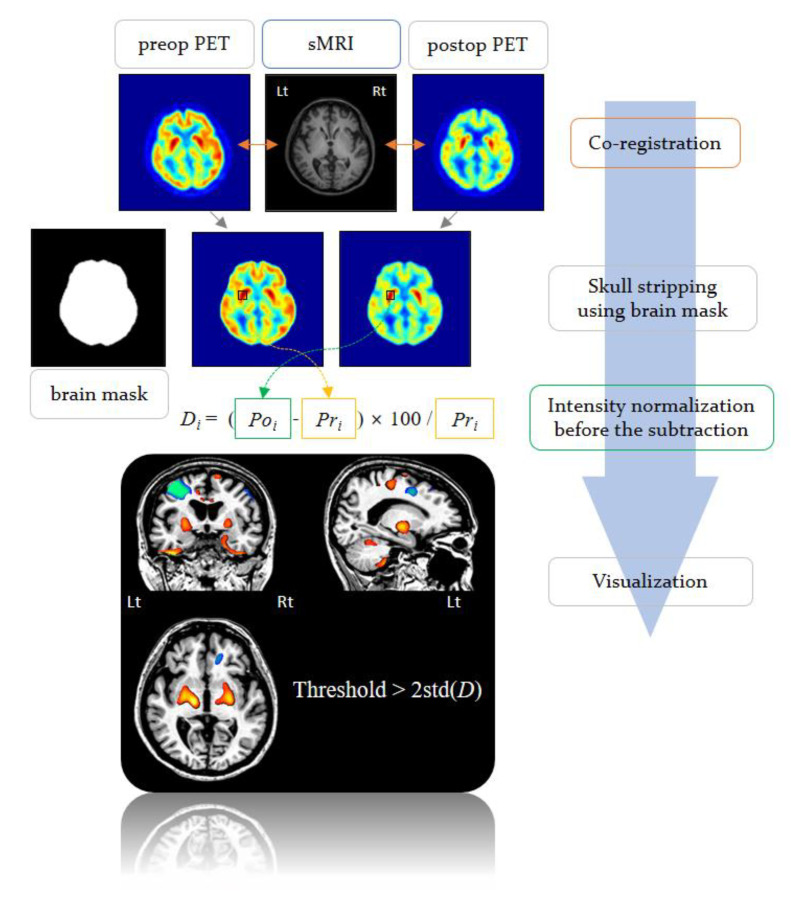
The process of voxel-based subtraction analysis of positron emission tomography (PET) images. In the present study, the threshold was set at 2 standard deviations (SD) from the mean value.

To investigate metabolic changes after DBS, a voxel-based subtraction analysis was performed by adopting the subtraction ictal SPECT co-registered to MRI (SISCOM) technique used in epilepsy (Hong S.B., 2014). The difference in radioisotope uptake levels was assessed by voxel-by-voxel subtraction of preoperative from postoperative ^18^F-FDG PET images [22]. This difference (*D_i_*) of a voxel (*i*) is defined as the subtraction of preoperative from postoperative ^18^F-FDG PET images given by

(1) $$D_{i}=\;\frac{\left(Po_{i}\;-\;Pr_{i}\right)}{Pr_{i}}\;\times 100\;\left(\%\right)$$

where *Po_i_* and *Pr_i_* denote the *i*-th voxel value of postoperative and preoperative ^18^F-FDG PET images, respectively. In the present study, the threshold was set at 2 standard deviations (SD) from the mean value. The subtraction image was represented as a distribution map of clustered and localized brain regions with differences in radioisotope uptake levels. Figure 4 describes the process of voxel-based subtraction analysis of PET in this study.

## 3. Results

### 3.1. Clinical Course

**Figure 5:**
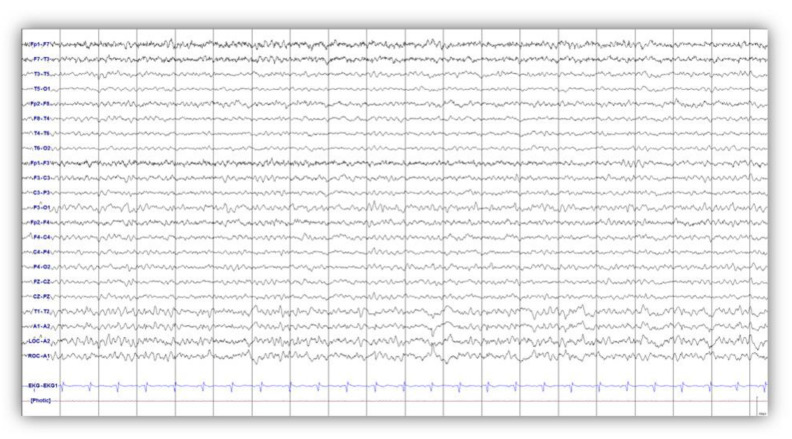
Electroencephalogram (EEG) performed five years after deep brain stimulation showed a reduced number and amplitude of slow-wave discharges, with no evidence of generalized spike or polyspikes from prefrontal or prefrontal seen in the previous EEG.

The initial programming consisted of a monopolar review (contact 0 and 1, amplitude 2 V, pulse width 90 µs, frequency 130 Hz). As the amplitude increased from 2 V to 3 V, the patient reported subjective weakness in her upper and lower extremities. Instead of changing the amplitude, the frequency was gradually increased from 130 Hz to 160 Hz. Both her rest and action myoclonus were aggravated, and were greater in her upper extremities (Supplementary Materials Video S3). Six weeks after DBS, we attempted to decrease the frequency to 40 Hz, and within a few minutes, reductions in both rest and action myoclonus were observed (Supplementary Materials Video S4). Three months after DBS electrode implantation, her unified myoclonus rating scale (UMRS) score decreased from 32 to 3 at rest, from 17 to 8 for stimulus sensitivity, and from 80 to 52 for action. From three months after DBS, the patient discontinued her topiramate treatment and took levetiracetam (1000 mg twice a day), valproic acid (500 mg twice a day), and clonazepam (1 mg twice a day). Nine months from the first programming session, a significant reduction in her action myoclonus was observed in both her arms and legs, without resting myoclonus. The negative myoclonus in her lower extremity also showed improvement, which allowed her to stand and walk using a walker. She could hold items with both hands and drink from a cup with one hand. The dose of one of her antiepileptic medications, clonazepam was reduced by 0.5 mg. Five years after DBS electrode implantation, she underwent an implantable pulse generator (IPG) replacement operation. The DBS parameters were adjusted to an amplitude of 2.5 V, a pulse width of 130 µs, and a frequency of 35 Hz. EEG performed five years after electrode implantation showed a reduced number and amplitude of slow-wave discharges, without generalized spikes or polyspikes with stimulation off (Figure 5). Although mild negative myoclonus remained in her lower extremities, she reported no aggravation or recurrence. She discontinued clonazepam and but continues levetiracetam (750 mg twice a day) and valproic acid (500 mg twice a day).

### 3.2. Voxel-Based Subtraction Analysis of PET 

**Figure 6:**
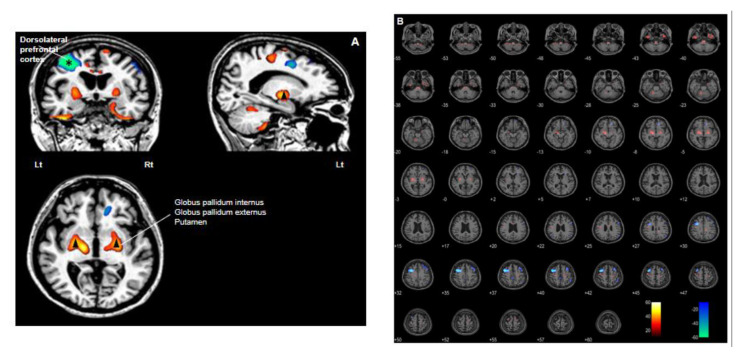
PET/MRI co-registration and voxel-based subtraction analysis of one year after deep brain stimulation (DBS) from pre-operation. Red scale indicates an increase in glucose metabolism and blue scale indicates a decrease in glucose metabolism. (**A**) A noticeable decrease in glucose metabolism in the dorsolateral prefrontal cortex (DLPFC) (asterisk) and an increase in glucose metabolism in the ventrolateral thalamus, globus pallidum internus (Gpi), globus pallidum externus (Gpe) and putamen (arrowhead). (**B**) A noticeable decrease in glucose metabolism was observed in the DLPFC, especially in the left hemisphere and an increase in glucose metabolism was identified in the medulla, pons, midbrain, cerebellar lobules, inferior temporal lobe, ventrolateral thalamus, Gpi, Gpe, putamen, and premotor cortex bilaterally on PET/MRI images obtained one year after DBS and compared with preoperative PET/MRI.

**Figure 7:**
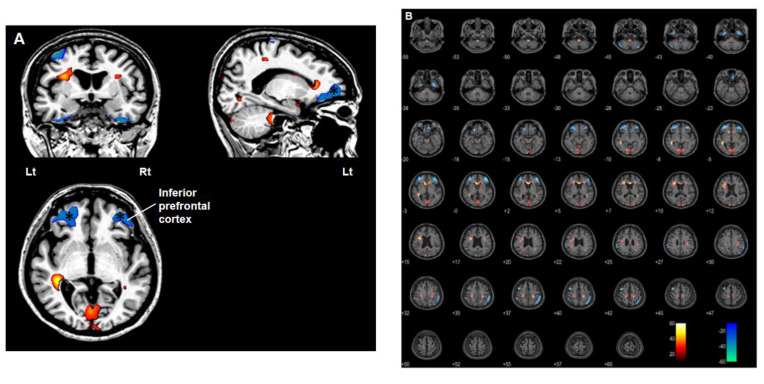
PET/MRI co-registration and voxel-based subtraction analysis of three years after deep brain stimulation (DBS) from pre-operation. Red scale indicates an increase in glucose metabolism and blue scale indicates a decrease in glucose metabolism. (**A**) A noticeable decrease in glucose metabolism in the orbitoprefrontal and inferior prefrontal cortex (asterisk). (**B**) A bilateral decrease in glucose metabolism was observed in the cerebellum, inferior temporal lobe, sensory cortex, superior parietal cortex and, especially orbito-, and inferior prefrontal cortex and an increase in glucose metabolism was identified in the medulla, pons, midbrain, occipital cortex including cuneus and calcarine sulcus, motor cortex mainly for leg and feet, anterior part of the insula, anterior cingulate cortex and genu of corpus callosum on the PET/MRI images obtained three years after DBS.

Using PET/MRI co-registration and voxel-based statistical parametric mapping to compare postoperative and preoperative images, one year after DBS we observed, bilaterally, a decrease in glucose metabolism in the dorsolateral prefrontal cortex (DLPFC), especially in the left hemisphere, and an increase in glucose metabolism in the medulla, pons, midbrain, cerebellar lobules, inferior temporal lobe, ventrolateral thalamus, Gpi, globus pallidus externus (Gpe), putamen, and premotor cortex (Figure 6). Table 1 presents the stereotactic coordinates and the peak value of each anatomical regions in SISCOM 1 year after DBS. Subsequently, our analysis of images obtained three years after DBS revealed a bilateral decrease in glucose metabolism in the cerebellum, inferior temporal lobe, sensory cortex, superior parietal cortex and, particularly, the orbito-/inferior prefrontal cortex, and an increase in glucose metabolism in the medulla, pons, midbrain, occipital cortex (including the cuneus and calcarine sulci), motor cortex (mainly areas concerned with motor activity of the legs and feet), anterior part of the insula, anterior cingulate cortex and genu of the corpus callosum (Figure 7). Table 2 present the stereotactic coordinates and the peak value of each anatomical regions in SISCOM 3 years after DBS. Based on these results, we hypothesize that interventions such as DBS that can modulate networks involving these structures may be useful in patients with severe PHM [9].

## 4. Discussion

### 4.1. Bilateral Gpi Deep Brain Stimulation for Post-Hypoxic Myoclonus

DBS has been suggested for patients with medically-refractory chronic PHM [4,14,15,16]. Our decision to choose the bilateral Gpi as the target for implantation was thus based on published data regarding the treatment of chronic PHM. However, there is a lack of published data regarding myoclonus treatment in PHM, and MD should be considered distinct from PHM in respect of the different pathogenesis. We referred to published data regarding the treatment of myoclonus with Gpi DBS in patients with MD [17,18,19,23]. Based on experience at our center, Kim et al. suggested that patients with MD may significantly benefit from bilateral Gpi DBS; the authors found optimal results at a long pulse width and high frequency [17]. Gruber et al. reported that combined and separate Gpi and ventral intermediate thalamic nucleus (VIM) DBS improved MD symptoms in 10 cases of incapacitating MD [18]. These patients required high-frequency stimulation to show myoclonus improvements. Liu et al. reported successful treatment of a patient with familial MD with DBS to the medial pallidum. The authors suggested that high-frequency DBS might suppress the myoclonus by desynchronizing abnormal pallidal oscillations [24,25]. In previous studies, PET results indicated that PHM is associated with changes in metabolic activity of the Gpi [26,27], especially because the Gpi is considered vulnerable to hypoxic brain damage [28,29]. It is known that globus pallidus (GP) neurons in the network decrease during high-frequency stimulation and increase during low frequency stimulation [30]. Although high-frequency stimulation targeting the Gpi has been reported in a few studies, in the present study, an increase in myoclonus ratings at high stimulation frequencies was observed, which is not in line with previous findings. When stimulation frequency was decreased, reductions in both rest and action myoclonus were observed. Low-frequency stimulation reduced the clinical myoclonus, which is also not consistent with the results of previous studies. While the mechanism underlying the improvement of myoclonus with low-frequency stimulation remains unclear, some studies proposed that low-frequency stimulation of the Gpi might also be effective for the treatment of dystonia [31,32,33,34,35]. Although a mean firing rate of the Gpi during an operation does not represent an optimal frequency of stimulation, Starr et al. demonstrated a mean firing rate of the Gpi of 50 Hz in intraoperative MER of Gpi neurons in patients undergoing pallidal surgery for dystonia [36], and Alterman et al. suggested that low-frequency stimulation may approximate the intraoperative firing rate of the target neurons [33]. The mean firing rate of the Gpi may vary from patient to patient. McClelland et al. reported MER data from Gpi in children undergoing DBS for dystonia and investigated differences in firing rates among the different types of dystonia. The authors identified that Gpi firing rates in the secondary group (median 9.6 Hz) showed significantly lower frequencies than the primary group (median 13.5 Hz) and among the secondary group, the hypoxic-ischemic encephalopathy (HIE) group in particular had significantly lower Gpi firing rates than those with later onset [37]. In the present study, myoclonus improved rapidly after low-frequency stimulation. Myoclonus is expected to disappear earlier than dystonic movement and posture [38], Chudy et al. also observed diminishing and disappearing myoclonism a few days after starting DBS of the central thalamic area [39]; however, rapid improvements after the introduction of low-frequency stimulation seem unusual. While only a blinded cross-over stimulation trial would allow us to draw clearer conclusions, we hypothesized that low-frequency stimulation might have induced an increase of Gpi firing rates which had been lowered after the hypoxic event. Therefore, activation of Gpi led to re-activation of GABA-mediated inhibitory postsynaptic mechanism in the frontal cortex [40].

### 4.2. Pathophysiology of Post-Hypoxic Myoclonus

The pathophysiology of PHM remains unknown. Initially, the pathophysiology of LAS was proposed to involve repetitive firing of the thalamocortical fibers arising from the ventrolateral nucleus of the thalamus, which is the main relay nucleus from the cerebellum to the sensorimotor cortex [3,41]. Although minimal anatomical changes were observed in recent human brain imaging studies on PHM, significant cortical and cerebellar connectivity, metabolic, and blood flow changes were found [9,10,42,43,44,45]. Notably, ^18^F-FDG PET findings by Frucht and colleagues revealed elevated glucose metabolism in the ventrolateral thalamus and pontine tegmentum in seven patients with PHM, indicating involvement of the basal ganglia-thalamocortical network. Based on the observation of hypermetabolism in the ventrolateral thalamus on PET scan, the authors suggested stereotactic targeting of the ventrolateral thalamus using DBS for selected patients with severe, medically refractory PHM [9]. However, this is not a consistent finding. Zhang et al. reported that the PET scan of a patient with LAS showed a mild bilateral decrease in glucose metabolism in the frontal lobes, compared to the scans of other patients [10]. Huang et al. conducted functional MRI (fMRI) of a PHM patient whose myoclonic jerks were more vigorous on the right leg than on the left. The authors identified increased activity in the bilateral cortex, especially the motor cortex of leg when the patient dorsiflexed her right foot compared with her left [45]. Another fMRI study on a patient with post hypoxic cortical myoclonus observed increased connectivity between the motor and sensory cortexes in the resting state [43]. fMRI has many potential advantages for studying functional brain connectivity and activity changes in neurobehavioral disorders [46], enabling us to investigate the pathogenesis of LAS. It is a non-invasive imaging technique with no radiation hazard. Besides artifacts from electrode, the clinical safety of 3-T MRI in patients with DBS implantation is not yet fully established, although there is some preliminary data showing that there was no heating, warmth, or adverse neurological effects in patients with implanted DBS systems who underwent 3-T MRIs [47]. Voxel-based subtraction analysis enables us to measure neuronal activity by exploiting glucose metabolism and identify functional neural changes after DBS implantation. Finding cerebral alterations that are not visible on conventional MRI is possible with an advanced PET analysis technique such as the one used in the present study. Co-registration of PET/MRI improved the anatomical definition of PET, and a voxel-based subtraction analysis enabled us to differentiate the functional neural changes after DBS on serial PET. It was not possible to compare the glucose metabolism of the brain before and after the hypoxic injury, since we did not assess ^18^F-FDG PET before the hypoxic event and therefore, did not have baseline measurements. In the present study, voxel-based subtraction analysis revealed an increase in glucose metabolism in the subcortical structures Gpi, Gpe and ventrolateral thalamus one year after DBS (Figure 6). Subsequently, we identified a significant reduction in glucose metabolism in the cerebral cortex, particularly, the bilateral prefrontal cortex three years after DBS (Figure 7). In the present study, we could not observe a definite evidence regarding the connectivity from the cerebellum to the cortex, although we observed a mild decrease in glucose metabolism in the cerebellum on PET/MRI performed 3 years after DBS. Results of studies on the pathogenesis of PHM vary, making it difficult to make a conclusion about its origin and the effect of DBS stimulation. According these results, we hypothesize that DBS may involve the stabilization of neuronal activity within the cortex which exhibits significant activation after hypoxic brain damage by stimulating the subcortical structure, Gpi.

### 4.3. Gpi DBS Reduced Glucose Metabolism in Prefrontal Cortex

The patient showed involuntary movements of both arms and legs in the resting state and precipitated by action (Video 1). EEG showed frequent bilateral generalized high-voltage polyspike discharges (Figure 1). These findings suggested that the origin of myoclonus in this patient might be the cortex, as epileptiform discharges such as spike or polyspike discharges suggest a cortical origin [43,48]. Furthermore, the polyspike discharges were recorded mainly in the bilateral prefrontal and frontal electrodes. We observed a decrease in glucose metabolism in the DLPFC in the voxel-based subtraction analysis performed one year after DBS and in the orbito-/inferior prefrontal cortex and DLPFC three years after DBS. We modulated DBS parameters and identified that low frequency stimulation was optimal, as the patient showed a significant reduction in her action myoclonus without resting myoclonus. EEG performed five years after DBS showed slow-wave discharges, with no evidence of generalized spike or polyspikes from prefrontal or frontal that had been seen in the previous EEG. A study investigating the effect of chronic Gpi-DBS on brain activity in focal and segmental dystonia revealed markedly reduced resting cerebral blood flow (rCBF) in the prefrontal cortex in patients who underwent Gpi-DBS for dystonia [49]. The mechanism of decreased activity in the prefrontal network in dystonia after Gpi DBS may be different from the decrease in glucose metabolism in the prefrontal cortex in PHM. PHM shares similarities with juvenile myoclonic epilepsy (JME), in terms of the clinical presentation of myoclonus. Koepp et al. suggested that JME is associated with the possible involvement of the DLPFC and thalamic dysfunction. Furthermore, the authors proposed that increased functional connectivity between the motor and prefrontal cognitive systems may occur in JME [50]. Rubboli et al. suggested that positive myoclonus involves in the primary motor cortex and negative myoclonus depends on the activation of cortical inhibitory areas [6]. Also, the author proposed that epileptic negative myoclonus was associated with a frontal cortical potential suggesting involvement of frontal areas in the generation of negative myoclonus [51]. Usui et al. observed iomazenil SPECT images, which reflect the specific binding of the tracers to GABA-A receptors of a patient with epileptic negative myoclonus exhibited significant decrease in the left medial frontal area [40]. The negative myoclonus might be associated with the supplement motor area (SMA). In the present study, we did not present a definite topography of myoclonus-related component, but we identified a decrease in glucose metabolism in the prefrontal lobe which was suspected to be the origin of the myoclonus according to the clinical presentation and EEG findings. However, we did not demonstrate an alteration in functional connectivity between the subcortical and cortical areas in our study. The structures that had been activated and deactivated after one year and three years following DBS were not coherent. An increase in glucose metabolism in the Gpi suggests that DBS may modulate pathological neural activity within stimulation sites [52,53], and that ongoing interference of cellular firing rates requires increased glucose metabolism; therefore, DBS results in a consuming state of activity [54]. Stimulation of the Gpi might induce activation of GABA-mediated inhibitory postsynaptic mechanism in the frontal cortex, triggering the stabilization of neuronal activity within the cortex which exhibits activation after hypoxic damage. Although PHM is a rare disorder, more cases and longitudinal studies are necessary to elucidate the underlying mechanism of PHM and the effect of Gpi DBS in terms of reducing pathological PHM-related activity, by potentially reorganizing subcortical-cortical circuits.

### 4.4. Limitations

The imaging analysis used in the present study has several limitations. Currently, due to improvements in voxel-based subtraction analysis, data from a healthy control group are used to define the distribution of FDG accumulation in normal structures with reference to anatomical MRI findings. However, in the present study, the regional abnormality of cerebral metabolic activity was not determined by comparing the patient’s scan with age-matched healthy controls selected from a data base [55]. Furthermore, standard uptake value ratios (SUVr) on ^18^F-FDG PET are quantitatively measured and used to compare SUVr for each voxel of interest between normal and pathologic structures [56]. Further studies on LAS using regional SUVr could help our understanding of metabolic changes correlating with functional outcomes and thus the pathophysiology of PHM and LAS. PHM is a rare disorder, as previously mentioned. As DBS is considered for patients with PHM in rare cases, it is difficult to confirm clinical evidence for the effectiveness of DBS for PHM on a case-by-case basis. We did not present a satisfying clinical evidence for meaningful effect of DBS for PHM, because spontaneous recovery of myoclonus in LAS after few years could often not be excluded [57]. We acknowledged limitations to clearly establish whether recovery is spontaneous or due to DBS. In addition, the longitudinal PET studies were performed only in the stimulation-on state. A voxel-based subtraction analysis with stimulation off after a washout period would have revealed the long-term effect of Gpi DBS on PHM and the functional changes in the patient due solely to DBS, excluding the possibility of spontaneous recovery and underlying pathologic changes from the hypoxic event itself. Not only the clinical improvement over time but also the changes in PET studies and EEG findings are not completely excluded from the possibility of outcomes resulting from the spontaneous remission of PHM. Further investigations including more cases are necessary to confirm whether DBS can contribute to the improvement of patients with PHM.

## 5. Conclusions

We present a patient with medically refractory PHM following cardiopulmonary arrest who experienced clinical improvements with bilateral Gpi DBS. We conclude from this case that when modulating DBS parameters for the treatment of PHM, decisions on adjusting stimulation parameters should be based on the patient’s clinical symptoms. Our experience suggests that low-frequency stimulation might be helpful to control myoclonus in PHM. Although it is difficult to confirm our hypothesis with a single-case observation, the present results indicate that voxel-based PET subtraction may be a useful approach for monitoring patients with PHM treated with DBS. Further investigations and continuous follow-up on the use of the advanced technique of PET analysis as well as DBS treatment for patients with PHM are necessary to help our understanding of the pathophysiology of PHM and LAS.

## Figures and Tables

**Table 1:** The values given are the stereotactic MNI coordinates and the peak value of each anatomical region. The threshold was set at 2 standard deviations of SISCOM results. (Voxel size: [2.0, 2.0, 2.0] mm, Minimum cluster size threshold of 50 voxels.).

	SISCOM 1 Year After Deep Brain Stimulation		
Anatomical Region	MNI Coordinates	Number of Voxels	Maximum Subtraction Value (%)
L Putamen	−28, −12, −2	167	19.38
R Putamen	28, −4, −2	60	16.62
L Pallidum	−26, −6, −4	110	20.77
R Pallidum	26, −8, −4	133	20.77
L Thalamus	−14, −12, −2	33	22.15
R Thalamus	14, −10, −2	6	16.62
L Middle Frontal Gyrus	−36, 12, 44	698	−48.46
R Middle Frontal Gyrus	32, 26, 52	259	−23.54
L Precentral Gyrus	−40, −2, 56	267	22.15
R Precentral Gyrus	24, −14, 56	7	16.62
L Midbrain	−14, −14, −8	98	30.46
R Midbrain	14, −10, −2	47	16.62
L Inferior Temporal Gyrus	−38, −6, −46	95	20.77
R Interior Temporal Gyrus	44, 4, −46	149	24.92

R, right; L, left; MNI, Montreal Neurological Institute, SISCOM, subtraction ictal SPECT co-registered to MRI.

**Table 2:** The values given are the stereotactic MNI coordinates and the peak value of each anatomical region. The threshold was set at 2 standard deviations of SISCOM results. (Voxel size: [2.0, 2.0, 2.0] mm, Minimum cluster size threshold of 50 voxels.).

	SISCOM 3 Years After Deep Brain Stimulation		
Anatomical Region	MNI Coordinates	Number of Voxels	Maximum Subtraction Value (%)
L InferiorTemporal Gyrus	−32, −8, −44	44	−42.92
R InteriorTemporal Gyrus	38, −4, −46	61	−34.61
L InferiorFronto-Orbital Gyrus	−34, 46, −16	95	−34.61
R InferiorFronto-Orbital Gyrus	48, 46, −4	132	−45.69
R Supramarginal Gyrus	58, −32, 44	180	−41.54
L Supramarginal Gyrus	−60, −28, 38	89	−41.54
R Postcentral Gyrus	50, −32, 50	17	−34.61
L Postcental Gyrus	−62, −20, 30	4	−30.46
R Cuneus	8, −94, 24	46	40.15
L Cuneus	−4, −94, 20	106	38.77
R Calcarine Sulcus	4, −70, 8	55	30.46
L Calcarine Sulcus	−2, −76, 6	119	34.61
R Insular	30, 28, 6	4	27.69
L Insular	−30, 28, 12	161	41.54
R Anterior Cingulate Cortex	2, 28, 0	4	33.23
L Anterior Cingulate Cortex	−8, 28, −4	41	48.46

R, right; L, left; MNI, Montreal Neurological Institute, SISCOM, subtraction ictal SPECT co-registered to MRI.

## Supplementary Materials

The following are available online at https://www.mdpi.com/2076-3425/10/10/730/s1, Video S1: She could not hold a cup because of her action myoclonus., Video S2: She needed assistance to stand, which triggered negative myoclonus in her legs, and the shock-like involuntary negative myoclonus caused postural lapses., Video S3: When the frequency was increased from 130 Hz to 160 Hz, both her rest and action myoclonus were aggravated., Video S4: When the frequency was decreased to 40 Hz, reductions in both rest and action myoclonus were observe.

## References

[1] Szczepanska A., Dziadkowiak E., Bladowska J., Kipiński L., Budrewicz S., Koszewicz M. (2019). The Usefulness of Quantitative EEG and Advanced MR Techniques in the Monitoring and Long-Term Prognosis of Lance-Adams Syndrome. Front. Neurol..

[2] Hallett M. (2000). Physiology of human posthypoxic myoclonus. Mov. Disord..

[3] Lance J.W., Adams R.D. (1963). The syndrome of intention or action myoclonus as a sequel to hypoxic encephalopathy. Brain.

[4] Ramdhani R.A., Frucht S.J., Kopell B.H. (2017). Improvement of Post-hypoxic Myoclonus with Bilateral Pallidal Deep Brain Stimulation: A Case Report and Review of the Literature. Tremor Other Hyperkinet. Mov. (NY).

[5] Tassinari C.A., Rubboli G., Shibasaki H. (1998). Neurophysiology of positive and negative myoclonus. Electroencephalogr. Clin. Neurophysiol..

[6] Rubboli G., Tassinari C.A. (2006). Negative myoclonus. An overview of its clinical features, pathophysiological mechanisms, and management. Neurophysiol. Clin..

[7] Shibasaki H. (1995). Overview and classification of myoclonus. Clin. Neurosci..

[8] Shibasaki H. (1995). Pathophysiology of negative myoclonus and asterixis. Adv. Neurol..

[9] Frucht S.J., Trost M., Ma Y., Eidelberg D. (2004). The metabolic topography of posthypoxic myoclonus. Neurology.

[10] Zhang Y.-X., Liu J.-R., Jiang B., Liu H.-Q., Ding M.-P., Song S.-J., Zhang B.-R., Zhang H., Xu B., Chen H.-H. (2007). Lance-Adams syndrome: A report of two cases. J. Zhejiang Univ. Sci. B.

[11] Fahn S. (1978). Post-Anoxic Action Myoclonus: Improvement with Valproic Acid. N. Engl. J. Med..

[12] Obeso J.A. (1995). Therapy of myoclonus. Clin. Neurosci..

[13] Wille C., Steinhoff B.J., Altenmüller D.-M., Staack A.M., Bilic S., Nikkhah G., Vesper J. (2011). Chronic high-frequency deep-brain stimulation in progressive myoclonic epilepsy in adulthood-Report of five cases. Epilepsia.

[14] Yamada K., Sakurama T., Soyama N., Kuratsu J. (2011). Gpi pallidal stimulation for Lance-Adams syndrome. Neurology.

[15] Kobayashi K., Katayama Y., Otaka T., Obuchi T., Kano T., Nagaoka T., Kasai M., Oshima H., Fukaya C., Yamamoto T. (2010). Thalamic Deep Brain Stimulation for the Treatment of Action Myoclonus Caused by Perinatal Anoxia. Ster. Funct. Neurosurg..

[16] Asahi T., Kashiwazaki D., Dougu N., Oyama G., Takashima S., Tanaka K., Kuroda S. (2015). Alleviation of myoclonus after bilateral pallidal deep brain stimulation for Lance–Adams syndrome. J. Neurol..

[17] Kim J.H., Na Y.C., Lee W.H., Chang W.S., Jung H.H., Chang J.W. (2014). Bilateral globus pallidus interna deep-brain stimulation in a patient with myoclonus-dystonia: A case report. Neuromodulation.

[18] Gruber D., Kühn A.A., Schoenecker T., Kivi A., Trottenberg T., Hoffmann K.-T., Gharabaghi A., Kopp U.A., Schneider G.-H., Klein C. (2010). Pallidal and thalamic deep brain stimulation in myoclonus-dystonia. Mov. Disord..

[19] Kurtis M.M., Luciano M.S., Yu Q., Goodman R.R., Ford B., Raymond D., Pullman S.L., Saunders-Pullman R. (2010). Clinical and neurophysiological improvement of SGCE myoclonus–dystonia with GPi deep brain stimulation. Clin. Neurol. Neurosurg..

[20] Kiebel S.J., Ashburner J., Poline J.-B., Friston K. (1997). MRI and PET Coregistration—A Cross Validation of Statistical Parametric Mapping and Automated Image Registration. NeuroImage.

[21] Lange C., Suppa P., Frings L., Brenner W., Spies L., Buchert R. (2015). Optimization of Statistical Single Subject Analysis of Brain FDG PET for the Prognosis of Mild Cognitive Impairment-to-Alzheimer’s Disease Conversion. J. Alzheimer’s Dis..

[22] Lung H.J., Weng Y.-H., Wen M.-C., Hsiao I.-T., Lin K.-J. (2018). Quantitative study of 18F-(+)DTBZ image: Comparison of PET template-based and MRI based image analysis. Sci. Rep..

[23] Ramdhani R.A., Frucht S.J., Behnegar A., Kopell B.H. (2016). Improvement of Isolated Myoclonus Phenotype in Myoclonus Dystonia after Pallidal Deep Brain Stimulation. Tremor Other Hyperkinet. Mov. (NY).

[24] Liu X., Griffin I.C., Parkin S.G., Miall R.C., Rowe J.G., Gregory R.P., Scott R.B., Aziz T.Z., Stein J.F. (2002). Involvement of the medial pallidum in focal myoclonic dystonia: A clinical and neurophysiological case study. Mov. Disord..

[25] Zhang Y.-Q., Wang J.-W., Wang Y.-P., Zhang X.-H., Li J.-P. (2019). Thalamus Stimulation for Myoclonus Dystonia Syndrome: Five Cases and Long-Term Follow-up. World Neurosurg..

[26] Kobayashi S., Momose T., Sakurai M., Kanazawa I. (2012). Postanoxic akinesia with bilateral pallidal lesions: A PET study. Intern. Med..

[27] Yoshii F., Kozuma R., Takahashi W., Haida M., Takagi S., Shinohara Y. (1998). Magnetic resonance imaging and 11C-N-methylspiperone/positron emission tomography studies in a patient with the interval form of carbon monoxide poisoning. J. Neurol. Sci..

[28] Bhatia K.P., Marsden C. (1994). The behavioural and motor consequences of focal lesions of the basal ganglia in man. Brain.

[29] Carella F., Grassi M.P., Savoiardo M., Contri P., Rapuzzi B., Mangoni A. (1988). Dystonic-Parkinsonian syndrome after cyanide poisoning: Clinical and MRI findings. J. Neurol. Neurosurg. Psychiatry.

[30] Fleming J.E., Lowery M.M. Changes in Neuronal Entropy in a Network Model of the Cortico-Basal Ganglia during Deep Brain Stimulation. Proceedings of the 2019 41st Annual International Conference of the IEEE Engineering in Medicine & Biology Society.

[31] Kim J.P., Chang W.S., Park Y.S., Chang J.W. (2012). Effects of relative low-frequency bilateral globus pallidus internus stimulation for treatment of cervical dystonia. Ster. Funct. Neurosurg..

[32] Kumar R., Dagher A., Hutchison W.D., Lang A.E., Lozano A.M. (1999). Globus pallidus deep brain stimulation for generalized dystonia: Clinical and PET investigation. Neurology.

[33] Alterman R.L., Miravite J., Weisz D., Shils J.L., Bressman S.B., Tagliati M. (2007). Sixty hertz pallidal deep brain stimulation for primary torsion dystonia. Neurology.

[34] Goto S., Mita S., Ushio Y. (2002). Bilateral pallidal stimulation for cervical dystonia. An optimal paradigm from our experiences. Ster. Funct. Neurosurg..

[35] Sarva H., Miravite J., Swan M.C., Deik A., Raymond D., Severt W.L., Kopell B.H. (2017). A Case of Myoclonus-Dystonia Responding to Low-frequency Pallidal Stimulation. Tremor Other Hyperkinet. Mov. (NY).

[36] Starr P.A., Turner R.S., Rau G., Lindsey N., Heath S., Volz M., Ostrem J.L., Marks W.J. (2006). Microelectrode-guided implantation of deep brain stimulators into the globus pallidus internus for dystonia: Techniques, electrode locations, and outcomes. J. Neurosurg..

[37] McClelland V.M., Valentin A., Rey H., Lumsden D.E., Elze M.C., Selway R., Alarcon G., Lin J.-P. (2016). Differences in globus pallidus neuronal firing rates and patterns relate to different disease biology in children with dystonia. J. Neurol. Neurosurg. Psychiatry.

[38] Cif L., Valente E.M., Hemm S., Coubes C., Vayssiere N., Serrat S., Di Giorgio A., Coubes P. (2004). Deep brain stimulation in myoclonus-dystonia syndrome. Mov. Disord..

[39] Chudy D., Deletis V., Almahariq F., Marčinković P., Škrlin J., Paradžik V. (2018). Deep brain stimulation for the early treatment of the minimally conscious state and vegetative state: Experience in 14 patients. J. Neurosurg..

[40] Usui K., Matsuda K., Terada K., Nikaido K., Matsuhashi M., Nakamura F., Umeoka S., Usui N., Tottori T., Baba K. (2010). Epileptic negative myoclonus: A combined study of EEG and [123I]iomazenil (123I-IMZ) single photon emission computed tomography indicating involvement of medial frontal area. Epilepsy Res..

[41] Waddell A., Dirweesh A., Ordonez F., Kososky C., Peddareddygari L.R., Grewal R.P. (2017). Lance–Adams syndrome associated with cerebellar pathology. J. Community Hosp. Intern. Med. Perspect..

[42] Carbon M., Raymond D., Ozelius L., Saunders-Pullman R., Frucht S., Dhawan V., Bressman S., Eidelberg D. (2013). Metabolic changes in DYT11 myoclonus-dystonia. Neurology.

[43] Park K.M., Han Y.H., Kim T.H., Mun C.W., Shin K.J., Ha S.Y., Park J., Kim S.E. (2015). Increased functional connectivity between motor and sensory cortex in a patient with Lance–Adams syndrome. Clin. Neurol. Neurosurg..

[44] Ferlazzo E., Gasparini S., Cianci V., Cherubini A., Aguglia U. (2013). Serial MRI findings in brain anoxia leading to Lance–Adams syndrome: A case report. Neurol. Sci..

[45] Huang H.-C., Chen J.-C., Lu M.-K., Tsai C.-H. (2010). Post-hypoxic cortical myoclonus mimicking spinal myoclonus-electrophysiological and functional MRI manifestations. Eur. J. Neurol..

[46] Filippi M., Elisabetta S., Piramide N., Agosta F. (2018). Functional MRI in Idiopathic Parkinson’s Disease. Int. Rev. Neurobiol..

[47] Sammartino F., Krishna V., Sankar T., Fisico J., Kalia S.K., Hodaie M., Kucharczyk W., Mikulis D.J., Crawley A., Lozano A.M. (2017). 3-Tesla MRI in patients with fully implanted deep brain stimulation devices: A preliminary study in 10 patients. J. Neurosurg..

[48] Dijk J.M., Tijssen M.A. (2010). Management of patients with myoclonus: Available therapies and the need for an evidence-based approach. Lancet. Neurol..

[49] Greuel A., Pauls K.A.M., Koy A., Südmeyer M., Schnitzler A., Timmermann L., Fink G.R., Eggers C. (2020). Pallidal Deep Brain Stimulation Reduces Sensorimotor Cortex Activation in Focal/Segmental Dystonia. Mov. Disord..

[50] Koepp M.J., Woermann F., Savic I., Wandschneider B. (2013). Juvenile myoclonic epilepsy–neuroimaging findings. Epilepsy Behav..

[51] Rubboli G., Parmeggiani L., Tassinari C.A. (1995). Frontal inhibitory spike component associated with epileptic negative myoclonus. Electroencephalogr. Clin. Neurophysiol..

[52] McIntyre C.C., Anderson R.W. (2016). Deep brain stimulation mechanisms: The control of network activity via neurochemistry modulation. J. Neurochem..

[53] Chiken S., Nambu A. (2016). Mechanism of Deep Brain Stimulation: Inhibition, Excitation, or Disruption?. Neuroscientist.

[54] Baldermann J.C., Bohn K.P., Hammes J., Schüller C.B., Visser-Vandewalle V., Drzezga A., Kuhn J. (2019). Local and Global Changes in Brain Metabolism during Deep Brain Stimulation for Obsessive-Compulsive Disorder. Brain Sci..

[55] Shin J.H., Kim Y.K., Kim H.J., Kim J.S. (2014). Altered brain metabolism in vestibular migraine: Comparison of interictal and ictal findings. Cephalalgia.

[56] Lee E.J., Oh J.S., Moon H., Kim M.-J., Kim M.S., Chung S.J., Kim J.S., Jeon S.R. (2019). Parkinson Disease-Related Pattern of Glucose Metabolism Associated With the Potential for Motor Improvement After Deep Brain Stimulation. Neurosurgery.

[57] Werhahn K.J., Brown P., Thompson P.D., Marsden C.D. (1997). The clinical features and prognosis of chronic posthypoxic myoclonus. Mov. Disord..

